# Analysis of Patient Factors Affecting In Vitro Characteristics of Subacromial Bursal Connective Tissue Progenitor Cells during Rotator Cuff Repair

**DOI:** 10.3390/jcm10174006

**Published:** 2021-09-04

**Authors:** Daichi Morikawa, Benjamin C. Hawthorne, Mary Beth R. McCarthy, Nicholas Bellas, Jeremiah D. Johnson, Maxwell T. Trudeau, Kyle V. Murphy, Michael R. Mancini, Matthew R. LeVasseur, Mark P. Cote, Augustus D. Mazzocca

**Affiliations:** 1 Department of Orthopaedic Surgery, University of Connecticut, Farmington, CT 06032, USA; bhawthorne@uchc.edu (B.C.H.); mccarthy@uchc.edu (M.B.R.M.); nbellas@uchc.edu (N.B.); jejohnson@uchc.edu (J.D.J.); trudeau@uchc.edu (M.T.T.); kymurphy@uchc.edu (K.V.M.); mimancini@uchc.edu (M.R.M.); mlevasseur@uchc.edu (M.R.L.); mcote@uchc.edu (M.P.C.); 2 Department of Orthopaedic Surgery, Juntendo University Urayasu Hospital, Urayasu 279-0021, Japan

**Keywords:** subacromial bursa, rotator cuff, mesenchymal stem cells, connective tissue progenitors, shoulder

## Abstract

Unsatisfactory failure rates following rotator cuff (RC) repair have led orthopaedic surgeons to explore biological augmentation of the healing enthesis. The subacromial bursa (SB) contains abundant connective tissue progenitor cells (CTPs) that may aid in this process. The purpose of the study was to investigate the influence of patient demographics and tear characteristics on the number of colony-forming units (CFUs) and nucleated cell count (NCC) of SB-derived CTPs. In this study, we harvested SB tissue over the supraspinatus tendon and muscle in 19 patients during arthroscopic RC repair. NCC of each sample was analyzed on the day of the procedure. After 14 days, CFUs were evaluated under a microscope. Spearman’s rank correlation coefficient was then used to determine the relationship between CFUs or NCC and patient demographics or tear characteristics. The study found no significant correlation between patient demographics and the number of CFUs or NCC of CTPs derived from the SB (*p* > 0.05). The study did significantly observe that increased tear size was negatively correlated with the number of CFUs (*p* < 0.05). These results indicated that increased tear size, but not patient demographics, may influence the viability of CTPs and should be considered when augmenting RCrepairs with SB.

## 1. Introduction

Rotator cuff (RC) pathology is one of the most common etiologies of shoulder dysfunction with more than 4.5 million physician visits annually [1]. The leading treatment choice following failure of conservative measures remains surgical repair of the torn tendon back to its humeral attachment. Day et al. reported that from 2007 to 2015, there was a 188% increase in total RC repairs in the United States [2]. Despite advancements in surgical material and techniques, retear rates following RC repairs approximate 10 to 94% depending on the patient population [3,4,5]. A major issue following repair of the tendon to bone interface is failed enthesis regeneration; instead of the enthesis regenerating to its native form, healing results in a mechanically inferior fibrous scar [6,7,8].

Several approaches for augmenting the healing tendon have been proposed and developed, including pharmacological, biological scaffolds and stem/progenitor cell transplantation [9,10,11]. “Stem cell” transplantation for tendon repair has been explored from many sources, including embryonic stem cells (ESCs), induced pluripotent stem cells (iPSCs), and adult stem cells such as mesenchymal stem cells (MSCs) and adipose-derived stem cells [12,13]. Currently, in the United States, the FDA does not permit ex vivo culture expansion of cells as Regulation 21 CFR 1271 requires products to be homologous, minimally manipulated, and lack systemic effects [14,15]. Thus, the majority of stem cell research has searched for local populations of MSCs that that can be utilized intraoperatively in an efficient manner for a low cost [11,14,16].

Previous literature has demonstrated that the subacromial bursa (SB) harbors a high concentration of cells that meet the International Society for Cellular Therapy (ISCT) criteria for MSCs [17,18,19,20,21]. When freshly harvested from patients, cells with characteristics of MSCs are more accurately described as connective tissue progenitors (CTPs) [11,13,22,23]. The bursal CTPs have been shown to have high proliferation/differentiation potential, as well as superior engraftment to host tendon in animal models [19,24,25,26,27]. Due to the ease of accessibility and inexpensive nature of harvesting the SB intraoperatively, surgical techniques have been developed to efficiently augment RC repairs with these CTPs [28,29,30]. Additionally, this process does not fall under the complex FDA regulations applied to that other “stem cell” therapies such as ESCs and iPSCs [14,31].

In order to determine which patients may be good candidates for biological augmentation during RCrepairs, it is important to understand how patient demographics alter characteristics of CTPs, as this may affect healing. Patients who are old, obese, diabetic, and smoke have previously been reported to have higher rates of RC repair failures compared to healthy patients [32,33,34,35]. Muench et al. was the first to demonstrate that SB CTPs demonstrated high proliferation potential regardless of patient demographics and RC tear characteristics [36]. The study was limited as it used nucleated cell count (NCC) as a proxy for cell proliferation, which is less specific for cellular activity of CTPs compared to colony-forming units (CFUs) [13,22,37].

The purpose of this study was to further investigate the influence of patient demographics and tear size on the number of CFUs and NCC of SB-derived CTPs. The authors hypothesized that patient demographics and tear size would not be correlated to proliferative characteristics of SB CTPs.

## 2. Materials and Methods

### 2.1. Patient Selection

Patients were selected for this study from December 2016 to May 2018. These individuals had to be undergoing arthroscopic RC repair to be considered for enrollment. Institutional review board approval was acquired before initiation of the study (IRB no. IE-07-224-2). Inclusion criteria for patients selected for the study included an age > 18 years and clinical indication for arthroscopic RC repair following the failure of alternative conservative therapy. Exclusion criteria included patients with a history of systemic infectious disease (sexually transmitted infections, Hepatitis infection, etc.) and patients who were pregnant, imprisoned, or deemed to be another form of a vulnerable population. Prior surgery to the affected shoulder was not cause for exclusion from the study. For every participant, basic demographic information (age, sex, height, weight, BMI), pertinent medical and surgical history, whether the patients smoked (never, former, current), and the duration they smoked (months) was collected. Smoking information was then used to determine a Brinkman index score based on cigarettes per day multiplied by years smoked to quantify the amount someone has smoked. The presence of diabetes mellitus, thyroid disease, and hypertension were also collected.

### 2.2. Surgical Removal of Subacromial Bursal Tissue

Harvest of SB was performed using techniques feasible in the setting of arthroscopic RC repair [25]. Bursal tissue samples were obtained using an arthroscopic grasper device in two locations in each patient for comparison, above the supraspinatus tendon and above the supraspinatus muscle. The samples were placed into sterile specimen cups filled with sterile normal saline. After collection, samples were immediately transported from the operating room to a laminar flow hood for processing.

### 2.3. Subacromial Tissue Processing

For each sample, 200 mg was carefully weighed for plating. As previously described, the sample was then digested in 2 mg/mL Collagenase P (Sigma-Aldrich, St. Louis, MO, USA) in Dulbecco’s modified Eagle’s medium (DMEM) (1× Thermo Fisher Scientific, Waltham, MA, USA) at 37 °C for 2 h [24,37]. Following digestion, the cells were filtered through a 70-mm cell strainer (Fisher Scientific, Pittsburgh, PA, USA), the remnant was disposed of and the cells were centrifuged at 800× *g* for 5 min to obtain a cell pellet [21].

### 2.4. Nucleated Cell Count

After digestion, the cellular concentrations (cells/mL) were counted using a Z1 Coulter Particle Counter (Beckman Coulter Life Sciences, Indianapolis, IN, USA), calibrated to detect particles > 8 mm, using a transparent cuvette containing 100 mL of cellular solution and 9.9 mL of 0.9% NaCl solution for a total volume of 10 mL. The number of nucleated cells was then calculated, using this cellular concentration, by multiplying by the volume of DMEM (10 mL). The cell mass density (cells/mg) was then calculated by normalizing with respect to the total mass of the harvested tissue.

### 2.5. Colony-Forming Units

After NCC, the cells were plated in complete culture medium containing DMEM, 10% fetal bovine serum (FBS) (Thermo Fisher Scientific), and 0.1% penicillin/streptomycin (Thermo Fisher Scientific) on Corning Primaria 100-mm dishes (Thermo Fisher Scientific) at a density of 10^3^ and 10^4^ for each bursa sample and were grown at 37 °C in a CO_2_ incubator at 5% humidified CO_2_ [19,21]. Complete DMEM medium was changed every 3 to 4 days. After 14 days in culture, dishes of each density were stained with 0.5% crystal violet solution for 10 min. The cells were then washed twice with distilled water, and the number of colonies per dish was counted. Colonies measuring < 2 mm in diameter or faintly stained colonies were not included [19,24,37].

### 2.6. Radiographic Analysis

All patients received magnetic resonance imaging (MRI) of the involved shoulder prior to surgery. MRI was utilized to determine tear size for each patient in the anteroposterior (AP) view. All MRIs were evaluated by two independent sports medicine trained orthopaedic surgeons.

### 2.7. Statistical Analyses

Continuous data were summarized using mean and standard deviation (SD), as well as median and interquartile ranges (IQR). Categorical variables were presented as frequencies and percentages. Spearman’s rank correlation coefficient was utilized to assess statistical correlations between patient demographics and the number of CFUs and NCC of colonies by SB CTPs. These analyses were completed for both bursa over the RC tendon and bursa over the RC muscle. All *p* values of <0.05 were considered statistically significant. Analyses were performed using Stata 17 (StataCorp LLC, College Station, TX, USA).

## 3. Results

### 3.1. Subjects

Nineteen patients underwent arthroscopic RC repair, performed by a single surgeon (ADM) from December 2016 to May 2018. The mean age of patients was 57.2 ± 4.5 years (median 57 years; IQR 54–60 years) and the majority of patients were female (*n* = 10; 52.6%). The mean weight was 92.5 ± 16.4 kg (median 88.5; IQR 82.6–102.9), the mean height was 169.5 ± 9.9 cm (median 167.6; IQR 161.3–176.5), and the mean BMI was 32.5 ± 5.5 kg/m^2^ (median 32.4; IQR 29.2–34.4). The mean tear size was 18.6 ± 9.3 mm (median 13.5; IQR 11.0–27.0). The majority of patients in the series (84.2%) had a history of smoking (never: *n* = 6, 31.6%; current: *n* = 10, 52.6%; former: *n* = 3, 15.8%), resulting in a mean Brinkman Index of 300.6 ± 386.8 (median 50.0; IQR 0–573.3). Lastly, regarding chronic comorbidities, no patients in the series had rheumatoid arthritis, 21.1% of the cohort had diabetes mellitus, 15.8% had thyroid disease, and 66.7% had hypertension.

### 3.2. Correlation between Number of CFUs and Patient Demographics

There were no statistically significant correlations between number of CFUs from SB CTPs over RC tendon or muscle and the following patient demographics: age, sex, height, weight, BMI, smoking, Brinkman Index, diabetes mellitus, thyroid disease, and hypertension. Further details can be observed in Table 1.

**Figure 1:**
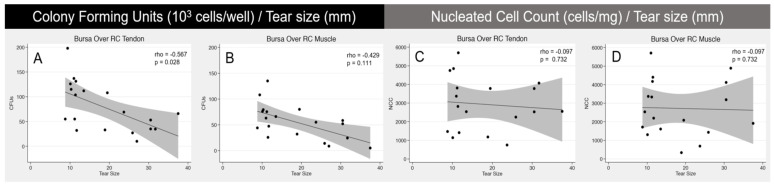
Scatterplot representations of Spearman’s rank correlation coefficient analysis. The left scatterplot demonstrates a significant (*p* < 0.028) negative correlation between tear size and number of CFUs from the cells of the SB over the RC tendon (**A**). The right three scatterplots show no significant correlation between tear size and CFU from SAB over RC muscle (**B**) or NCC of the cells from SB over RC tendon (**C**) and muscle (**D**). Grey shading around regression line represents 95% confidence interval.

Notably, tear size (mm) was the only variable that demonstrated a significant correlation with number of CFUs by cells from SB over the RC tendon. Focusing on bursa over the RC tendon, tear size was significantly correlated with number of colonies when analyzing 10^3^ cells/well (rho = −0.567; *p =* 0.028) and 10^4^ cells/well (rho = −0.645; *p* = 0.009). Scatterplot representation of the Spearman’s rank correlation coefficient (rho) between tear size and CFU number can be observed in Figure 1.

### 3.3. Correlation between NCC and Patient Demographics

When analyzing the correlation between NCC from SB CTPs over the RC tendon and muscle (Table 2), no statistical correlation was seen with any patient demographics. This analysis included age, sex, height, weight, BMI, tear size, smoking, Brinkman Index, diabetes mellitus, thyroid disease, and hypertension.

## 4. Discussion

The most important finding of this study was that there was no significant correlation between patient demographics and the number of CFUs or NCC of CTPs derived from the SB over the RC tendon and muscle. Additionally, this study demonstrated a significant negative correlation between increased tear size and the number of CFUs from bursa over RC tendon.

With unsatisfactory retear rates and a long postoperative rehabilitation period [4,38,39,40,41], orthopaedic surgeons have begun augmenting RC tears with the readily accessible SB [28,29,30]. Recent literature has demonstrated that the SB is an abundant source of CTPs that meet the criteria set by the ISCT, including adherence to plastic culture plates, the expression of specific cell surface molecules, and multi-lineage differentiation [17,18,19,20,21]. In vitro studies have further demonstrated the high proliferative potential of these cells [19,24,25,26,27]. Although bone-marrow aspirate has long been utilized as a source of CTPs for regenerative orthopaedic surgery [22,23,42,43,44], studies now show that bursal CTPs have significantly higher proliferation, CFUs, and differentiation ability compared to bone-marrow MSCs [24]. Additionally, Dyrna et al. demonstrated that bursal CTPs showed superior engraftment and survival in tendon tissue than bone-marrow MSCs [27]. These results have led to the development of multiple inexpensive techniques to augment RC repairs using the SB with minimal manipulation [28,29,30].

Specific patient demographics and comorbidities have long been associated with RC repair failure, such as advancing age [45,46], smoking [38,47,48,49,50], diabetes mellitus [51,52,53,54], and obesity [33,55,56], among others [41,57]. When deciding which patients may be good candidates for bursal biological augmentation of RC repairs, these demographics may concern orthopaedic surgeons that their tissue may not be viable. Muench et al. were the first to demonstrate that patient demographics and RC tear characteristics did not alter the proliferation potential of bursal CTPs [36]. The study was limited in that they used NCC, a nonspecific measure of cellular activity that cannot differentiate live, dead, or other mononuclear cells. CTPs are defined partly by their ability to divide to form a clonal population, known as CFUs, which may be a more clinically relevant measure of CTP viability [13,22]. The present study demonstrated that the number of CFUs was not correlated to patient demographics, further supporting Muench et al.’s conclusion that the SB may be used for biological augmentation regardless of patient demographics [36].

This study is limited as the in vitro nature of CTPs may not mimic the in vivo potential of CTPs in the shoulder. Further studies are needed to understand the relationship between CFUs and the in vivo interaction of CTPs with the healing tendon. Additionally, the study’s small sample size (*n* = 19) may result in insufficient statistical power and possible type II error.

Nevertheless, the results of the present study may further ease concerns that certain patient demographics could alter the characteristics of SB CTPs. This tissue complex is easily accessible during RC repair and, for a low cost, may be efficiently used to augment RC repairs. Future clinical trials are needed to understand the effects of SB CTPs on healing and outcomes of RC repairs.

## Figures and Tables

**Table 1:** Spearman’s rank correlation coefficient analysis of the number of Colony-Forming Units (CFUs) by cells from SB over the RC tendon and muscle. Correlation analysis between the number of CFUs and patient demographics was completed. While tear size was negatively correlated to the number of CFUs from both the cells from the SB over the RC tendon, there was no statistical correlation with other demographics. (* = significance *p* < 0.05).

	Number of Colony Forming Units							
	Bursa over Rotator Cuff Tendon				Bursa over Rotator Cuff Muscle			
	10^3^ Cells/Well		10^4^ Cells/Well		10^3^ Cells/Well		10^4^ Cells/Well	
	rho	*p*-Value	rho	*p*-Value	rho	*p*-Value	rho	*p*-Value
Age	−0.179	0.522	−0.319	0.246	−0.036	0.899	−0.013	0.965
Sex (Female)	0.000	1.000	−0.062	0.827	−0.031	0.913	0.186	0.508
Height (cm)	0.097	0.732	0.138	0.624	0.116	0.680	−0.091	0.746
Weight (kg)	−0.000	1.000	−0.173	0.537	0.136	0.629	−0.127	0.652
BMI (kg/m^2^)	−0.189	0.499	−0.368	0.177	−0.129	0.648	−0.161	0.567
Tear size (mm)	−0.567	0.028 *	−0.645	0.009 *	−0.429	0.111	−0.434	0.106
Smoking (Non, Former, Current)	−0.147	0.602	−0.137	0.627	0.221	0.429	−0.018	0.950
Brinkman Index (Ciggarettes/d × y)	0.113	0.690	0.105	0.709	0.270	0.331	0.094	0.739
Diabetes mellitus (Yes)	−0.232	0.407	−0.077	0.785	−0.424	0.115	0.039	0.891
Thyroid disease (Yes)	−0.347	0.205	−0.424	0.115	−0.193	0.491	−0.270	0.330
Hypertension (Yes)	0.196	0.483	0.196	0.483	0.223	0.411	0.196	0.483

**Table 2:** Spearman’s rank correlation coefficient analysis of nucleated cell count (NCC) from the SB over RC tendon and muscle. There was no statistical correlation with all patient demographics and tear characteristics.

	Nucleated Cell Count per Milligram			
	Bursa Over RC Tendon		Bursa over RC Muscle	
	rho	*p*-Value	rho	*p*-Value
Age	0.165	0.557	−0.040	0.889
Sex (Female)	0.000	1.000	0.000	1.000
Height (cm)	−0.091	0.746	−0.138	0.624
Weight (kg)	−0.363	0.184	−0.038	0.894
BMI (kg/m^2^)	−0.361	0.187	−0.057	0.840
Tear size (mm)	−0.097	0.732	−0.097	0.732
Smoking (Non, Former, Current)	−0.445	0.096	−0.078	0.782
Brinkman Index (Ciggarettes/d × y)	−0.299	0.280	0.028	0.922
Diabetes mellitus (Yes)	0.039	0.891	0.077	0.785
Thyroid disease (Yes)	−0.039	0.891	−0.270	0.330
Hypertension (Yes)	−0.229	0.411	−0.295	0.2865

## Data Availability

The data are not publicly available due to concerns over patient privacy. Data available on request.
